# The De Novo Genome Sequencing of Silver Pheasant (*Lophura nycthemera*)

**DOI:** 10.1093/gbe/evab275

**Published:** 2021-12-14

**Authors:** Xue-Juan Li, Xiao-Yang Wang, Chao Yang, Li-Liang Lin, Le Zhao, Xiao-Ping Yu, Fu-Min Lei, Yuan Huang

**Affiliations:** 1 College of Life Sciences, Shaanxi Normal University, Xi’an, China; 2 School of Biological and Environmental Engineering, Xi'an University, China; 3 Shaanxi Institute of Zoology, Xi’an, China; 4 School of Biological Sciences and Engineering, Shaanxi University of Technology, Hanzhong, China; 5 Key Laboratory of the Zoological Systematics and Evolution, Institute of Zoology, The Chinese Academy of Sciences, Beijing, China

**Keywords:** *Lophura nycthemera*, PacBio sequencing, genome assembly

## Abstract

Silver pheasant (*Lophura nycthemera*) belongs to Phasianidae, Galliformes, which exhibits high subspecific differentiation. In this study, we assembled a novel genome based on 98.42 Gb of Illumina sequencing data and 30.20 Gb of PacBio sequencing data. The size of the final assembled genome was 1.01 Gb, with a contig N50 of 6.96 Mb. Illumina paired-end reads (94.96%) were remapped to the contigs. The assemble genome shows high completeness, with a complete BUSCO score of 92.35% using the avian data set. A total of 16,747 genes were predicted from the generated assembly, and 16,486 (98.44%) of the genes were annotated. The average length of genes, exons, and introns were 19,827.53, 233.69, and 1841.19 bp, respectively. Noncoding RNAs included 208 miRNAs, 40 rRNAs, and 264 tRNAs, and a total of 189 pseudogenes were identified; 116.31 Mb (11.47%) of the genome consisted of repeat sequences, with the greatest proportion of LINEs. This assembled genome provides a valuable reference genome for further studies on the evolutionary history and conversion genetics of *L. nycthemera* and the phylogenomics of the Galliformes lineage.

Significance

The silver pheasant (*Lophura nycthemera*) is one of the least known pheasants of the world with a highly subspecific divergence. The high-quality reference genome assembly and annotation of *L. nycthemera* revealed several evolutionary features. This article provided a basic reference genome for facilitating studies on genomic characteristics and genome-based population divergence of *L. nycthemera* and phylogenomics of all pheasants of the world.

## Introduction

The development of high-throughput sequencing technology represented the beginning of a new era of genomic studies (Giordano et al., 2017), involving platforms such as Illumina, Pacific, and Nanopore sequencing. Genome sequences of birds, such as *Gallus gallus* (e.g., International Chicken Genome Sequencing Consortium, 2004), *Pseudopodoces humilis* (Qu et al., 2013), and *Zosterops lateralis* (Cornetti et al., 2015), facilitated by sequencing technologies, provide important information on avian evolution. Through comparative genomic analyses among Galliformes, some significant features have been found, such as characteristics related to high-altitude adaptation (Wang et al., 2015; Lee et al., 2018; Cui et al., 2019) and the coloration and pigmentation of plumage (Gao et al., 2018; Dhar et al., 2019), in addition to genetic and evolutionary characteristics (Jiang et al., 2019; Zhou et al., 2019). Recently, several Galliformes genomes have been described in GenBank database, which species covered all five families. Although long-read sequencing platform has been used to obtain Galliformes genomes, most of Galliformes genomes were also sequenced by using Illumina HiSeq sequencing technology.

Silver pheasant (*Lophura nycthemera*) (Phasianidae, Galliformes) is widely distributed in southern China, eastern Myanmar, northern Thailand, and the Indo–China Peninsula (Cheng et al., 1978), with a forest canopy coverage preference ranging from altitudes of 0–1,000 m (BirdLife International, Chen et al., 2019). It exhibits 15 subspecies, nine of which occur in China (Johnsgard 1999; Gill and Donsker, 2020). The plumage pattern of the upper parts of the males and topographic barriers are used to establish its taxa and relationships (Delacour 1948). Females are smaller than males, with polygamous lifestyles (Grimmett et al., 1999). For *L. nycthemera*, some subspecies with limited ranges exhibit potential conservation problems due to habitat loss and other influences (McGowan and Garson, 1995).

To study the basic genomics of *L. nycthemera* and explore the evolution of all Galliformes, we performed the de novo genome sequencing by combining the Illumina and PacBio platforms. In addition, based on the assembled results, we also studied its genomic features. This study provides a high-quality genome assembly of *L. nycthemera*, and will be helpful for further studying evolutionary features of Galliformes species.

## Results and Discussion

### Genome Assembly and Completeness Assessment

In this study, approximately 98.42 Gb of raw sequencing data were obtained from Illumina platform, with a sequencing depth of 93.73× (supplementary table S1, Supplementary Material online). The PacBio sequencing platform generated ∼30.20 Gb of raw data. A total assembly of 1.01 Gb with a contig N50 of ∼6.96 Mb was obtained. The genome size was similar to that of some other Galliformes species, such as 1.09 Gb of *Arborophila rufipectus* (Zhou et al., 2019). The contig number, contig length, contig N90, contig max, and GC content of the assembly genome were 1,553, 1,014,408,745 bp, 643,154 bp, 23,586,999 bp, and 41.38%, respectively.

**Fig. 1.**
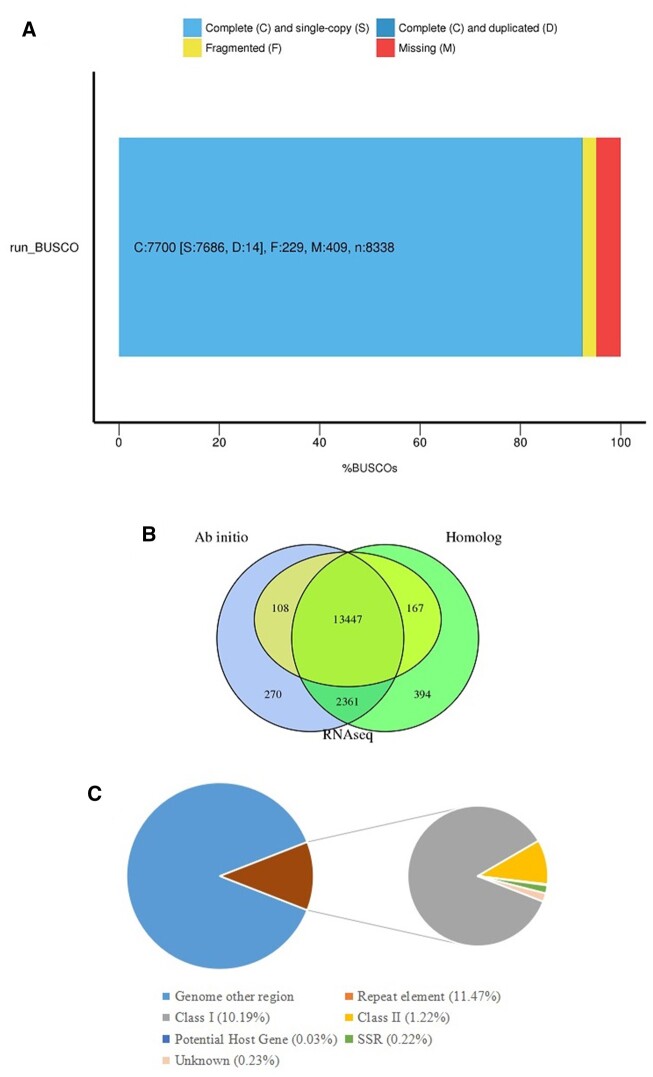
Statistics of genome assembly assessment, gene structure prediction and repeat element percentage of *Lophura nycthemera*. (*A*) BUSCO assessment results. (*B*) Predict results of gene structures using ab initio-based, homologue-based, and RNA-seq-based methods. (*C*) Percentage of repeat elements.

To assess assembled results, Illumina paired-end reads were remapped to the assembled genome, and 94.96% reads could be mapped to the contigs (supplementary table S2, Supplementary Material online). In addition, a total of 7,700 complete BUSCOs (92.35%) were identified in the assembly (fig. 1*A*). These results showed that the assembled genome was complete and presented a low error ratio. The complete and single-copy BUSCOs (92.18%) was higher than that of *A. rufipectus* (86.5%) (Zhou et al., 2019). The high-quality reference genomes of *L. nycthemera* could be a useful tool to understand genomic evolution.

### Gene Prediction and Functional Annotation

The consensus gene set contained 16,747 genes. The lengths of the genes, exons, CDS, and introns were shown in supplementary table S3, Supplementary Material online. The averages of gene length, exon length, and intron length were 19,827.53, 233.69, and 1841.19 bp, respectively. A total of 13,447 (80.29%) genes were supported by all three methods (fig. 1*B*), which represented a good gene prediction effect. A total of 16,486 (98.44%) predicted genes were successfully annotated by using nine databases (supplementary table S4, Supplementary Material online). The noncoding RNAs included 208 miRNAs, 40 rRNAs, and 264 tRNAs, which belonged to 100, 4, and 23 families, respectively. In addition, a total of 189 pseudogenes were identified.

### Repeat Sequences Annotation

It was estimated that 116.31 Mb (11.47%) of the genome consisted of repeat sequences (fig. 1*C* and supplementary table S5, Supplementary Material online). The percentage of repeat sequences was larger than those of other Galliformes species, such as 9.02% in *A. ardens* (Zhou et al., 2019) and 9.82% *G. gallus* (Dhar et al., 2019). Within Class I, the lengths of LINEs and SINEs sequences were 74 and 0.2 Mb, with percentages of 7.30% and 0.02%, respectively (supplementary table S5, Supplementary Material online). The LINEs represented the greatest proportion of the genome, which was also found in other avian genomes, such as *Pavo cristatus* (Dhar et al., 2019). Within Class II, 11,656,729 bp (1.15%) of TIR sequences were identified (supplementary table S5, Supplementary Material online).

## Materials and Methods

### Sampling and Sequencing

A *L. nycthemera* female species was collected from captive breeders in Lantian, Xi’an, Shaanxi Province, China in 2016. The muscle tissues were used for sequencing. DNA was extracted by the CTAB method, with DNA concentrations and quality measured by a NanoDrop 2000 system and a Qubit Fluorometer. Total RNA was extracted using TRIzol, with RNA concentrations measured by a NanoDrop 2000 system and an Agilent 2100 Bioanalyzer.

The Illumina HiSeq X-Ten and PacBio Sequel pipelines were used for genome sequencing. For the Illumina platform, five short-fragment paired-end libraries, including three of 270 bp and two of 350 bp, were constructed via Illumina sequencing. The genomic DNA was randomly fragmented by using the ultrasonic method, and target fragments were then filtered. A small-fragment sequencing library was constructed through a series of steps, including end repair, the addition of A and adaptor sequences, target fragment selection, and PCR. The size and quality of the libraries were detected by using an Agilent 2100 system and Q-PCR. Illumina double-ended sequencing with PE = 150 was applied. For the PacBio platform, long-fragment libraries were constructed. The DNA samples were sheared by using g-TUBE, and DNA damage was then repaired and end-repaired. Dumbbell-type adapters were ligated using exonuclease digestion. For the sequencing libraries, target segment selection was performed using BluePippin.

The Illumina HiSeq X-Ten pipeline was also used to obtain RNA sequences. For RNA fragment libraries, rRNA was isolated from total RNAs, and then fragmented randomly. The first-strand cDNA was synthesized using random hexamer primers by employing the fragmented rRNA-depleted RNA as a template. The second-strand cDNA was synthesized using DNA polymerase I and RNase H. After end-repair, A-tail, adaptor ligation, and purification, PCR amplification was conducted.

### Genome Assembly and Assessment

After filtering low-quality and short length reads from the PacBio data, Wtdbg2 (Ruan and Li, 2020) was used for assembly. Pilon was used to correct this assembly results by using Illumina data with three times. Two methods were employed to assess assembled results, that is, Illumina paired-end reads remapped to the assembled genome, and BUSCO v4 databases (Waterhouse et al., 2018) with aves_odb09 employed.

### Repetitive Sequence Annotation

The database of repeat sequences was constructed using structure-based and ab initio-based strategies, employing LTR-FINDER v1.05 and RepeatScout v1.05. This database was classified by PASTEClassifier, and merged with the Repbase database into a final database of repeat sequences. RepeatMasker v4.0.6 (Tarailo‐Graovac and Chen, 2009) was used to predict repeat sequences.

### Gene Prediction and Function Annotation

Three strategies were employed to predict gene structures, including ab initio-based, homologue-based and RNA-seq-based methods. Genscan, Augustus v2.4, GlimmerHMM v3.0.4, GeneID v1.4, and SNAP (version 2006-07-28) were used for ab initio-based prediction. GeMoMa v1.3.1 was employed for homologue-based prediction, mainly employing six species (*G. Gallus*, *Meleagris gallopavo*, *Taeniopygia guttata*, *Ficedula albicollis*, *Parus major*, and *Coturnix japonica*). Hisat v2.0.4 and Stringtie v1.2.3 were used for assembly based on the referenced RNA-seq data. TransDecoder v2.0 and GeneMarkS-T v5.1 were used for predicting genes. PASA v2.0.2 was used to predict for assembled unigene sequences based on RNA-seq data without references. In addition, EVMv1.1.1 (Haas et al., 2008) was used to integrate the above prediction results, and PASA v2.0.2 was employed for modification.

For ncRNAs, microRNAs and rRNAs were predicted through genome alignment using Blastn, employing the Rfam database. TRNAscan-SE v1.3.1 was used to predict tRNAs. For pseudogenes, based on GenBlastA v1.0.4 alignment, homologous gene sequences were searched in the genome. GeneWise v2.4.1 was employed to search for premature stop codons and frame shifts, and to identify pseudogenes.

To assign gene functions, we aligned the genes to nine functional databases by using BLAST v2.2.3, with *E*-value = 1e-5. The databases included COG, GO, KEGG, KOG, Pfam, Swissprot, TrEMBL, eggNOG, and NR.

## Supplementary Material

Supplementary data are available at *Genome Biology and Evolution* online.

## Supplementary Material

evab275_Supplementary_Data
