# Supplementary material for: The De Novo Genome Sequencing of Silver Pheasant (Lophura nycthemera)
Source: Genome Biol Evol. 2021 Dec 14;13(12):evab275. doi: 10.1093/gbe/evab275 (PMC8691047; doi:10.1093/gbe/evab275)
Supplement: evab275_Supplementary_Data [file evab275_supplementary_data.doc]

**Supplementary Material**

**Table S1.** Sequencing data produced by Illumina sequencing platform.

| Library | Data (Gb) | Depth (×) | Q20 (%) | Q30 (%) |
| --- | --- | --- | --- | --- |
| 270bp_1 | 17.10 | 16.29 | 95.66 | 90.28 |
| 270bp_2 | 23.89 | 22.75 | 95.96 | 90.79 |
| 270 bp_3 | 21.27 | 20.25 | 95.57 | 90.02 |
| 350 bp_1 | 18.13 | 17.27 | 95.88 | 91.30 |
| 350 bp_2 | 18.03 | 17.17 | 95.61 | 90.78 |
| Total | 98.42 | 93.73 | --- | --- |

**Table S2. The results of Illumina clean reads mapped into contigs.**

| Total reads | Mapped reads | Mapped (%) | Properly mapped reads | Properly mapped (%) |
| --- | --- | --- | --- | --- |
| 661,104,638 | 647,980,017 | 98.01 | 624,229,822 | 94.96 |

**Table S3**. The statistic results of gene information.

| Item | value |
| --- | --- |
| Gene number | 16,747 |
| Gene length (bp) | 332,051,569 |
| Average gene length (bp) | 19,827.53 |
| Exon length (bp) | 40,870,834 |
| Average exon length (bp) | 233.69 |
| Exon number | 174,895 |
| Average exon number | 10.44 |
| CDS length | 27,819,213 |
| Average CDS length (bp) | 162.26 |
| CDS number | 171,445 |
| Average CDS number | 10.24 |
| Intron length (bp) | 291,180,735 |
| Average intron length (bp) | 1841.19 |
| Intron number | 158,148 |
| Average intron number | 9.44 |

**Table S4**. Statistics of gene functional annotation.

| Database | Annotated number | Percentage (%) |
| --- | --- | --- |
| COG | 5,303 | 31.67 |
| GO | 9,314 | 55.62 |
| KEGG | 10,424 | 62.24 |
| KOG | 11,523 | 68.81 |
| Pfam | 14,480 | 86.46 |
| Swissprot | 11,796 | 70.44 |
| TrEMBL | 16,364 | 97.71 |
| eggNOG | 9,673 | 57.76 |
| NR | 16,392 | 97.88 |
| All | 16,486 | 98.44 |

**Table S5**. Repeat elements of the assembled genome.

| Type | Number | Length (bp) | Percentage (%) |
| --- | --- | --- | --- |
| ClassI | 481,063 | 103,368,478 | 10.19 |
| ClassI/DIRS | 610 | 39,416 | 0.00 |
| ClassI/LARD | 104,044 | 19,143,132 | 1.89 |
| ClassI/LINE | 315,834 | 74,093,054 | 7.30 |
| ClassI/LTR/Copia | 945 | 542,884 | 0.05 |
| ClassI/LTR/Gypsy | 7,336 | 1,386,774 | 0.14 |
| ClassI/LTR/Unknown | 47,537 | 19,139,628 | 1.89 |
| ClassI/PLE | 2,632 | 675,045 | 0.07 |
| ClassI/SINE | 1,467 | 201,453 | 0.02 |
| ClassI/TRIM | 329 | 415,936 | 0.04 |
| ClassI/Unknown | 329 | 30,657 | 0.00 |
| ClassII | 58,376 | 12,363,809 | 1.22 |
| ClassII/Crypton | 505 | 165,892 | 0.02 |
| ClassII/Helitron | 1,149 | 85,404 | 0.01 |
| ClassII/MITE | 135 | 15,738 | 0.00 |
| ClassII/Maverick | 693 | 63,912 | 0.01 |
| ClassII/TIR | 49,005 | 11,656,729 | 1.15 |
| ClassII/Unknown | 6,889 | 507,453 | 0.05 |
| PotentialHostGene | 578 | 306,995 | 0.03 |
| SSR | 2,964 | 2,272,307 | 0.22 |
| Unknown | 8,955 | 2,336,295 | 0.23 |
| Total | 551,936 | 116,310,577 | 11.47 |
